# Transposable elements and viruses as factors in adaptation and evolution: an expansion and strengthening of the TE-Thrust hypothesis

**DOI:** 10.1002/ece3.400

**Published:** 2012-10-16

**Authors:** Keith R Oliver, Wayne K Greene

**Affiliations:** 1 School of Biological Science and Biotechnology, Faculty of Science and Engineering, Murdoch UniversityPerth, W.A., 6150, Australia; 2 School of Veterinary and Biomedical Sciences, Faculty of Health Sciences, Murdoch UniversityPerth, W.A., 6150, Australia

**Keywords:** Adaptive Potential, evolutionary potential, holobiont, symbiosis, TE-Thrust, transposable element

## Abstract

In addition to the strong divergent evolution and significant and episodic evolutionary transitions and speciation we previously attributed to TE-Thrust, we have expanded the hypothesis to more fully account for the contribution of viruses to TE-Thrust and evolution. The concept of symbiosis and holobiontic genomes is acknowledged, with particular emphasis placed on the creativity potential of the union of retroviral genomes with vertebrate genomes. Further expansions of the TE-Thrust hypothesis are proposed regarding a fuller account of horizontal transfer of TEs, the life cycle of TEs, and also, in the case of a mammalian innovation, the contributions of retroviruses to the functions of the placenta. The possibility of drift by TE families within isolated demes or disjunct populations, is acknowledged, and in addition, we suggest the possibility of horizontal transposon transfer into such subpopulations. “Adaptive potential” and “evolutionary potential” are proposed as the extremes of a continuum of “intra-genomic potential” due to TE-Thrust. Specific data is given, indicating “adaptive potential” being realized with regard to insecticide resistance, and other insect adaptations. In this regard, there is agreement between TE-Thrust and the concept of adaptation by a change in allele frequencies. Evidence on the realization of “evolutionary potential” is also presented, which is compatible with the known differential survivals, and radiations of lineages. Collectively, these data further suggest the possibility, or likelihood, of punctuated episodes of speciation events and evolutionary transitions, coinciding with, and heavily underpinned by, intermittent bursts of TE activity.

## Introduction

The importance of transposable elements (TEs) to stress responses and adaptation was first proposed by Barbara McClintock who was also the discoverer of TEs (McClintock 1956, 1984). Since then much groundbreaking work has substantiated the view that TEs play a significant role in evolution (Georgiev 1984; Syvanen 1984; Finnegan 1989; Brosius 1991; McDonald 1993; Kidwell and Lisch 1997; Fedoroff 1999; Shapiro 1999; Bennetzen 2000; Bowen and Jordan 2002; Jurka 2004; Kazazian 2004; Biémont and Vieira 2006; Volff 2006; Wessler 2006; Feschotte and Pritham 2007; Muotri et al. 2007; Beauregard et al. 2008; Böhne et al. 2008; Hua-Van et al. 2011; Werren 2011). Building on this body of work, we have proposed TEs as powerful facilitators of evolution (Oliver and Greene 2009a) and have subsequently gone further than others by formalizing this general concept into an explicit, comprehensive, predictive, and testable hypothesis, which we call the “TE-Thrust hypothesis” (Oliver and Greene 2011). The basis of the TE-Thrust hypothesis is that TEs are powerful facilitators of evolution that can act to generate genetic novelties in both an active mode and a passive mode. Active mode: by transposition, including the exaptation of TE sequences as promoters, exons, or genes. Passive mode: when present in large homogeneous populations, TEs can cause ectopic DNA recombination resulting in genomic duplications, deletions or rearrangements (including karyotypic changes). Fecund lineages, those with many species (e.g., rodents and bats, which together make up 60% of mammals), are generally rich in viable (i.e., capable of activity) and active TEs, whereas nonfecund lineages (e.g., monotremes) have mainly nonviable (i.e., incapable of activity) and inactive TEs. Evolutionary transitions, for example, the evolution of the higher primates and evolutionary innovations, such as the mammalian placenta, also appear to be facilitated by TEs (Oliver and Greene 2011). An outline of the TE-Thrust Hypothesis is:

Many eukaryote lineages are able to tolerate some sacrifices in the present, that is, a genomic “load” or population, of mostly controlled, but possibly fitness-reducing TEs. Such lineages may, thereby, fortuitously, gain a continuum of “intra-genomic potential” whose extremities are conveniently described as “adaptive potential” and “evolutionary potential.” This intragenomic potential may be realized in the present, and/or in the descendant lineage(s) of the future. Note that this does not imply any “aim” or “purpose” to evolution, or any ability of evolution to “see” into the future.

As environmental or ecological factors change, or the lineages adopt new habitats, these intragenomic potentials can be realized. For example, adaptive potential can be realized to give small adaptive changes within a lineage, over short periods of time, such as the evolution of insecticide resistance, when insecticides become prevalent in the environment. Evolutionary potential can be realized, over much longer periods of time, perhaps in adaptive radiations, as in some rodents or bats.

At least some unicellular eukaryotic organisms do not appear to tolerate a genomic load of TEs (Galagan and Selker 2004; Pritham 2009), which suggests that TE-Thrust does not operate in all extant biological lineages. However, it is noteworthy that most eukaryotic species known to lack TEs are intracellular parasites with small genomes, including members of the *Babesia*, *Cryptosporidium*, and *Plasmodium* genera (Pritham 2009). This could be due to selection for small cell size and/or because the genomic plasticity engendered by TEs may not provide a net advantage to nonfree-living organisms that exist within a stable environment.

## TE-Thrust and Punctuated Equilibrium

Eldredge and Gould (1972) posed the concept of punctuated equilibrium from studies of the fossil record, as opposed to the then prevailing concept of phyletic gradualism. There is now independent support for punctuated equilibrium from studies of extant taxa (Cubo 2003; Pagel et al. 2006; Mattila and Bokma 2008; Laurin et al. 2012), from co-evolution (Toju and Sota 2009), and in extant and ancient genomes of *Gossypium* species due to intermittent TE activity (Palmer et al. 2012). TE-Thrust provides an intragenomic explanation of punctuated equilibrium (Oliver and Greene 2009a,b, 2011), as has also been suggested by Zeh et al. (2009), via epigenetic changes, and/or endogenization of retroviruses, in response to stress, and Parris (2009), via endogenization of retroviruses and environmental change.

The actual processes of speciation events seem to be poorly understood, but new species are said to emerge from many differing and rare single events (Venditti et al. 2010). However, two almost essential components seem to be necessary: reproductive isolation and intragenomic variation. Of these, intragenomic variation can be readily supplied by the hypothesized TE-Thrust (Oliver and Greene 2011), and reproductive isolation can be provided by a variety of means, including karyotypic changes, polyploidy, hybridization, and physical environmental or ecological factors (Venditti et al. 2010).

Much TE activity (active TE-Thrust) is thought to occur in intermittent bursts that interrupt more quiescent periods of low activity (Bénit et al. 1999; Marques et al. 2005; Cantrell et al. 2005; Pritham and Feschotte 2007; de Boer et al. 2007; Ray et al. 2008; Zeh et al. 2009; Erickson et al. 2011). These punctuation events can occur especially after intermittent infiltrations or amplifications of TEs. New acquisitions of TEs can be due to:

- Intermittent horizontal transposon transfer (HTT) (Schaack et al. 2010). This appears to be relatively rare, and probably tends to occur more often with some DNA-TEs, LTR retro-TEs, and the Bov-B LINE.
- The *de novo* synthesis of chimeric elements, for example, the hominid specific SVA (Wang et al. 2005). This is probably rare.
- The *de novo* syntheses of various SINEs, the younger ones (<100 Myr) of which are lineage specific (Piskurek et al. 2003; Kramerov and Vassetzky 2011). This is probably rare.
- Intermittent endogenizations of various RNA viruses (Bénit et al. 1999; Belyi et al. 2010; Horie et al. 2010). This may be relatively common, especially in mammals.
- Hybridization, especially in angiosperms (Michalak 2010). This appears to be common.
- Intermittent *de novo* modifications to successive families of TEs (e.g. L1 LINEs). This is relatively common.

An example of an intermittent burst is the L1 LINE in ancestral primates, where among a large number of overlapping families, L1PA6, L1PA7, and L1PA8 were apparently amplified intensively around 47 Mya. This seemingly contributed to a very large Alu SINE, and retrocopy, amplification at this time (Ohshima et al. 2003). TEs can result in the acceleration of the evolution of genes in a myriad of ways (Böhne et al. 2008; Goodier and Kazazian 2008; Hua-Van et al. 2011), providing a means for rapid species divergences in the affected lineages.

## Modes of TE-Thrust

All the hypothesized modes of TE-Thrust shown below are consistent with the data tabulated in Oliver and Greene (2011), but are expressed herein in different ways. All of them refer only to the potential for adaptation or evolution due to the hypothesized TE-Thrust. As other facilitators of evolution will possibly also be active in addition to TE-Thrust, and as environmental and ecological factors can frequently change, all these hypothesized capabilities of TE-Thrust need to be predicated by “if all else is equal”. These modes of TE-Thrust are extremes of continuums, so intermediate modes must occur.

Mode 1. Evolutionary potential may be realized, in concert with, or following, significant intermittent bursts of TE activity, in viable and heterogeneous TE populations, whether large or small. This can underlie what we designate as “Type I” punctuated equilibrium (stasis with punctuation events), due to intermittent active TE-Thrust.

Mode 2. Evolutionary potential may be realized, in concert with, or following, significant bursts of TE activity, in large viable and homogenous TE populations. This can result in what we designate as “Type II” punctuated equilibrium (gradualism with punctuation events) due to both ongoing TE-Thrust (largely passive), and to intermittent active TE-Thrust. If the TE population is small, then only intermittent active TE-Thrust is likely to occur as per mode 1.

Mode 3. Nonviable heterogeneous TE populations, whether large or small, may result in evolutionary stasis, due to a lack of both active and passive TE-Thrust.

Mode 4. If a nonviable TE population is both large and homogeneous, and not too degraded by mutations, then gradualism type evolution may occur, due largely to passive TE-Thrust. If the TE population is small, then little TE-Thrust is likely to occur as per mode 3.

## An Expansion of the TE-Thrust Hypothesis

Herein, the TE-Thrust hypothesis is further expanded from its original formulation. We acknowledge that in addition to TE-Thrust, other nongenomic facilitators of evolution may play a part in radiations and evolution, such as dynamic external factors, including geological, environmental, and ecological changes. Such factors may result in fragmentation of populations into small local demes, or larger disjunct sub-populations, which can result in reproductive isolation with possible divergence into novel taxa (Wright 1931; Eldredge 1995; Jurka et al. 2011). In addition to alleles drifting to fixation or extinction in demes, TE families likely also do so (Jurka et al. 2011) and we are in agreement with this. Additionally, in TE-Thrust we hypothesize that novel TEs as described above, may very occasionally be introduced into, or arise within, some demes or disjunct populations, but not into others, ultimately causing evolutionary transitions or the evolution of new taxa. We view the carrier subpopulation (CASP) hypothesis (Jurka et al. 2011) to be complementary to TE-Thrust, as it is about the fixation of TEs in populations and the details of mechanisms, or origins, of speciation, which were previously not included in the TE-Thrust hypothesis. The CASP hypothesis gains some support from the cotton genus (*Gossypium*) specific *Gorge* retro-TEs (Palmer et al. 2012), as *Gorge* seems to have spread to fixation in a small progenitor population of *Gossypium*. Indeed, both hypotheses are in agreement in strongly relating TEs to speciation and evolution. However, we suggest that karyotypic changes due to TE presence and activity, are among the factors that produce the reproductive isolation necessary for speciation, although we agree that geographic isolation into demes, niche availability, and many other phenomena (e.g., pheromone changes in insects) are also important factors.

We note that adaptive evolution via natural selection is, but one of the forces of evolutionary change. Other important forces, all of which are nonadaptive, comprise mutation, recombination, and random genetic drift (Lynch 2007). As TE-Thrust emphasizes a key intragenomic role for TEs in mutation and recombination, it fits comfortably with a growing body of evidence indicating that a significant portion of evolutionary changes are not adaptive in nature, but result from the accumulation of mildly deleterious mutations that can become fixed by genetic drift in populations of relatively small size (Fernández and Lynch 2011). Indeed, although the occasional highly deleterious TE insertion will be rapidly culled by purifying selection, TE insertions can themselves be viewed overall as an accumulation of neutral to mildly deleterious mutations that are subject to genetic drift. Activation of TEs, for example, during stress, or horizontal transfer of TEs etc., provides powerful complements to genetic drift. Thus, TEs accumulate by nonadaptive processes and can underpin nonadaptive change, and they also readily provide the raw material for future beneficial traits capable of undergoing positive selection.

We recognize that there are many known genomic facilitators of evolution, besides TE-Thrust. A few apposite examples are: symbiosis (Ryan 2007, 2009); hybridization (Ryan 2006; Larsen et al. 2010); noncoding RNA (Heimberg et al. 2008; Mattick 2011); horizontal gene transfer (Richards et al. 2006); whole genome duplications (Hoffmann et al. 2012), and viral driven evolution (Villarreal 2005, 2009; Ryan 2007; Villarreal and Witzany 2010; Feschotte and Gilbert 2012). Some facilitators of evolution may have greater importance in some clades than in others. For example, whole genome duplication (polyploidy) is apparently quite important in the evolution of angiosperms (Soltis et al. 2004). Ryan (2006) includes several of the examples above under the general descriptor “genomic creativity”.

## Horizontal Transfer of TEs in TE-Thrust

Mobile DNA has been classified into Class I retro-TEs (e.g., LTR elements, LINEs, and SINEs), and Class II DNA-TEs, composed of subclasses 1 (e.g., *Tc1-Mariner* and *hAT*) and 2 (*Helitron* and *Maverick*), as have been described and reviewed elsewhere (Wicker et al. 2007; Böhne et al. 2008; Goodier and Kazazian 2008; Kapitonov and Jurka 2008; Hua-Van et al. 2011). The horizontal transfer of TEs (horizontal transposon transfer or HTT) has previously been proposed as a major force driving genomic variation and biological innovation (Schaack et al. 2010). DNA-TEs have long been known to be capable of HTT, for example, the *P*-element DNA-TE in *Drosophila* (Anxolabéhère et al. 1988; Daniels et al. 1990); the *Mariner* DNA-TE in various insects (Maruyama and Hartl 1991; Robertson and Lampe 1995; Lampe et al. 2003), and DNA-TEs in the bat *Myotis lucifugus* (Pritham and Feschotte 2007; Ray et al. 2007). However, HTT of retro-TEs, has been less well documented, except for some examples, including the patchily distributed Bov-B LINE, (Kordiš and Gubenšek 1998; Gogolevsky et al. 2008) and the Gypsy-like retro-TEs (Herédia et al. 2004).

Although probably infrequent, HTT is an important aspect of the TE-Thrust hypothesis that has so far only been given cursory attention (Oliver and Greene 2009a, 2011). Over 200 cases of HTT have been documented (Schaack et al. 2010), 12 of which were between different phyla. About a half of these known HTTs involved retro-TEs, most of which were LTR retro-TEs. The remaining HTTs involved a variety of DNA-TEs. Horizontal transfer is an important part of the life cycle of TEs, as they generally accumulate mutations and eventually become nonviable in the genomes they occupy. This can downgrade the efficacy of TE-Thrust. However, they are sometimes enabled, via chance events, to periodically make fresh starts with fully functional elements, in the genomes of other lineages. At least some TEs appear to be able to endure in the absence of HTT. For example, the LINE 1 (L1) retro-TE in mammals has persisted for 100 Myr with no known evidence of HTT (Furano et al. 2004; Khan et al. 2006), although it has now become nonviable in a few mammalian lineages (Casavant et al. 2000; Boissinot et al. 2004; Cantrell et al. 2008; Platt and Ray 2012).

Viruses and bacteria appear to be likely vectors of HTT (Piskurek and Okada 2007; Schaack et al. 2010; Dupuy et al. 2011), but endoparasites and intracellular parasites are among other possible vectors that have been proposed (Silva et al. 2004; Schaack et al. 2010). Empirical data (Anxolabéhère et al. 1988; Cantrell et al. 2005; de Boer et al. 2007; Pritham and Feschotte 2007; Ray et al. 2008) and simulations (Le Rouzic and Capy 2005) both suggest that TE amplification occurs immediately after HTT of a viable TE copy.

## Holobionts and Holobiontic Genomes, and The Importance of the Highly Mobile Retroviruses

Exogenous retroviruses can become endogenized, and can be united with the host genome into a holobiontic genome in a new holobiont (Box 1). Holobiont is a symbiological term that means the partnership, or union, of symbionts (Rosenberg et al. 2007; Ryan 2007; Gilbert et al. 2010). For example, the *ERVWE1* locus in the human genome comprises a conserved *envelope (env)* gene together with the conserved 5′ LTR of a retrovirus that contains regulatory elements. This locus, additionally, includes sections of human genetic sequences and these also play a role in regulation of the *env* gene, which codes for *Syncytin-1* (Mi et al. 2000). Syncytin-1 has a crucial function in trophoblast cell fusion in ape placental morphogenesis (Mi et al. 2000), which strongly suggests that selection has occurred at the level of the holobiontic genome in the human plus retrovirus holobiont (Ryan 2006).

**Box 1. Glossary of Terms**

**Parasite and Symbiont:** To most contemporary biologists, a parasite is an often harmful organism in a partnership that benefits itself at the expense of the other partner, and a symbiont is an organism in a mutually beneficial partnership with another organism. However, **Symbiologists** define **Symbiosis** as: The living together of differently named (i.e., different species) organisms, including **parasitism**, **commensalism,** and **mutualism** (Ryan 2006, 2009) and this definition is used here. **TE-Thrust:** A hypothesized pushing force generated by TEs within genomes, that can facilitate adaptation, and punctuated or major evolution, within the corresponding lineages (Oliver and Greene 2011). **Virus:** Viruses are a part of biology because they possess genes, have group identity, replicate, evolve, and are adapted to particular hosts, biotic habitats, and ecological niches. Most viruses are persistent and unapparent, that is, not pathogenic (Villarreal 2005). **Viral Biogenesis:** Exogenous retroviruses, and some other exogenous RNA viruses, can act in mutualism when endogenized in other genomes, and their genomes are united with the host genome into a “holobiontic genome”. **Holobiont:** The partnership, or union, of symbionts (Ryan 2007; Gilbert et al. 2010). **Mobilome:** A general term for the total content of the mobile DNA in any genome. **Mobilome Consortium** (Villarreal) implies that the presence or activity of each individual or category of TE, within the Mobilome, likely affects the mobilome as a whole, e.g., SINE viability is coupled to LINE compatibility and viability. **Adaptive potential:** The potential of a lineage to adapt over decades or centuries. Such adaptation can be associated with one to several genes. **Evolutionary potential:** The potential of a lineage to evolve and radiate, possibly by punctuation events, over thousands or millions of years. Such evolution may be associated with major organizational and architectural genomic changes. Note: Adaptive potential and Evolutionary potential are not distinct entities, but are useful descriptors for the extremities of an **Intra-genomic potential** continuum.

Retroviruses appear to be the most mobile of all “mobile DNA” as they can exist exogenously as infectious, or persisting viruses, as well as by becoming endogenized in host germ lines (Hughes and Coffin 2001, 2004; Ryan 2006). Exogenous retroviruses are distinct entities to those species whose genomes into which they endogenize to become an ERV, and they have an extracellular or virion stage, with a protein capsid. ERVs then are a part of a holobiont organism. Other TEs in a genome are not considered to be a part of a holobiont, as they seemingly can only transfer from genome to genome, and can have no independent existence like that of an exogenous retrovirus species.

Endogenized retroviruses (ERVs) can multiply within a genome either by repeated endogenizations, or by retrotransposition within the genome (Belshaw et al. 2004; Wang et al. 2010). Over time, due to recombinations between their LTRs, and deletions, ERVs often exist mostly as solo LTRs or sLTRs, (Sverdlov 1998). Many Class I elements are related to retroviruses, namely the *Copia, Gypsy,* and *BEL/Pao* subclasses of LTR retro-TEs, which have LTRs (long- terminal repeats), but lack an *env* gene.

Retroviruses are present among all placental mammals (Bénit et al. 1999), are largely restricted to vertebrates, and are particularly abundant in mammals (Villarreal 2005). Retroviruses have been endogenized in mammalian germ lines many times during the evolution of mammals. These ERVs have been a very important factor in their evolution (Villarreal 2005), and are particularly associated with that mammalian innovation, the placenta (Oliver and Greene 2011). Endogenized retroviruses, and the role they play in evolution, have been extensively detailed elsewhere (Villarreal 1997, 2004, 2005, 2009; Ryan 2003, 2006, 2007; Feschotte and Gilbert 2012).

Endogenous nonretroviral RNA virus elements, notably Bornaviruses, have also been found in mammalian genomes, including several primates and several rodents, and these viral sequences appear to have function (Belyi et al. 2010; Horie et al. 2010). Indeed, all major types of eukaryotic viruses can give rise to endogenous viral elements or EVEs (Feschotte and Gilbert 2012). Thus, viral-eukaryote holobiont organisms appear to be not uncommon, and these could have lead to significant evolutionary innovation. This enhances the explanatory power of the TE-Thrust hypothesis.

## Retroviruses and the Evolution of the Mammalian Placenta

The placenta represents a major evolutionary innovation that occurred over 160 Mya at the time of the divergence of the placental mammals. The circulatory and the metabolic benefits provided by this transient organ to the growing embryo and fetus have been well investigated, but less so well understood is the origin of the placenta. The invasive syncytial plate, the precursor to the placenta, and the rapidly growing trophoblast, are developmentally unique to mammals (Harris 1991). Harris proposes that prior to the divergence of placental mammals, developing embryos became infected at an early intrauterine stage with retroviruses, which gave rise to cellular proliferation and creation of the trophoblast. This may then have resulted in the formation of the highly invasive “tumor-like” vacuolated and microvillated syncytial plate and a primitive placenta (Harris 1991). Although to date, there is no proof that the fusogenic ERVs of premammals resulted in the evolution of the mammalian placenta (Harris 1991; Dupressoir et al. 2009) it seems likely to be correct. Supporting evidence comes from the egg-laying platypus, which has a genome that is devoid of ERVs, although there are some thousands of ancient *Gypsy*-class LTR retro-TEs (Warren et al. 2008). In contrast, all examined placental mammal genomes do contain many ERVs (Mayer and Meese 2005; Villarreal 2005), with ERV/sLTRs constituting approximately 8% and 10% of the human and mouse genomes, respectively (Waterston et al. 2002). Atypically, the placenta exhibits global DNA hypomethylation, which allows many ERVs and retro-TEs to retain transcriptional activity in this tissue (Rawn and Cross 2008). Such a permissive environment for expression of TEs facilitates their exaptation as coding or regulatory sequences, and indeed, the LTRs of ERVs contain promoter activity that can confer tissue-specific expression in the placenta, as for example, the *CYP19A1*, *IL2RB*, *NOS3*, and *PTN* genes, which are solely expressed by an LTR promoter (Cohen et al. 2009). Although there are few known unique placenta-specific genes, numerous genes expressed in the human placenta are derived from retro-TEs and ERVs (Rawn and Cross 2008). Most notable are the fusogenic, ERV *env*-derived, syncytin-1, and syncytin-2 (Mi et al. 2000; Blaise et al. 2003), with syncytin-2 also having an immunosuppressive function (Kämmerer et al. 2011). The efficient adaptive immune systems of mammals must fail to initiate an immune reaction to the antigens of their embryos and placentas, and mammals alone are very highly infected with the generally immunosuppressive endogenous retroviruses (Villarreal 1997). Intriguingly, retroviruses are abundant around sperm heads and also coat the female placenta (Steele 2009). The advantages of the placenta could possibly explain why extant placental mammals number well over 5,000 species, whereas there are less than 300 extant species of marsupials (Pough et al. 2009).

## Evolvability and the TE-Thrust Hypothesis

Mutation, including gene duplication and other DNA changes, is the driving force of evolution at both the genic and the phenotypic levels (Nei 2005, 2007). Significantly, Shapiro (2010) proposes that it is mobile DNA movement, rather than replication error that is the primary engine of protein evolution. Along the same lines, Hua-Van et al. (2011) stress TEs as a major factor in evolution, whereas Muotri et al. (2007) proposes that “handy junk” can evolve into “necessary junk”. Wagner (Heard et al. 2010), in support of our original concepts (Oliver and Greene 2009a) states that, in general, “the kinds of genetic changes that are possible depend on what kinds of TEs are present and active at any particular time”, in the evolution of each lineage. Thus, the potential for evolutionary innovations differs over time, contradicting the concept of gradualism in lineages. Caporale (2009) posits that “selection must act on the mechanisms that generate variation, much as it does on beaks and bones”. Earl and Deem (2004), with no mention of TEs, propose the evolution of mechanisms to facilitate evolution, and describe evolvability as a selectable trait. Further to this, Woods et al. (2011) found experimental evidence, in a study of bacteria that long-term evolvability may be important for determining the ultimate success of a lineage, and that less fit lineages with greater evolvability may eventually out-compete lineages with greater fitness. All these lines of reasoning, and associated experimental data, are in good accord with the TE-Thrust hypothesis.

## Reduced “Fitness” versus enhanced “Adaptive Potential” and “Lineage Selection”

Accumulation of TEs in the genome of *Drosophila melanogaster* has been found to be associated with a decrease in fitness (Pasyukova et al. 2004). The reduced “fitness” in *Drosophila* may be an extreme case, because in *D. melanogaster* TEs cause over 50% of *de novo* mutations (Pasyukova et al. 2004). In contrast to *D. melanogaster*, *de novo* disease-causing insertions in humans are relatively rare (Deininger and Batzer 1999; Kazazian 1999; Chen et al. 2005; Hedges and Batzer 2005), whereas TE activity in the laboratory mouse falls between these two extremes (Kazazian 1998; Waterston et al. 2002; Maksakova et al. 2006). There is, however, no conflict with the TE-Thrust hypothesis with this finding in *Drosophila*, as despite a fitness loss in some individuals in the present, there can be a fortuitous gain in adaptive potential to the lineage as a whole. TEd-alleles (TE- deactivated or destroyed alleles), for example, usually lower the fitness of the lineage. However, TEm-alleles (TE-modified alleles, which can be modified in either regulation or function, or duplicated), for example, increase the genetic diversity, and hence the adaptive potential, of the lineage. These TEm-alleles allow the lineage to adapt to environmental/ecological challenges in the present. Also, importantly, this adaptive potential may be latent in the present, and only be realized in the future, as environmental/ecological challenges change. This latent adaptive potential then, increases the chances of the long-term survival of the lineage. In other words, TE-Thrust can result in latent adaptive potential (also called standing variation), which can be realized, if needed, in the future, and can result in the differential survival of lineages. This is the rationale for positing lineage selection in the TE-Thrust hypothesis (Oliver and Greene 2009a,b, 2011).

## Realizable “Adaptive Potential” Due to TE-Thrust

TE-Thrust is proposed to have facilitated adaptive change, as we highlighted in the simian lineage (Oliver and Greene 2011). The ongoing ability of TEs to provide realizable adaptive potential is illustrated by TE-generated polymorphic traits identified in isolated populations of laboratory-bred mice (Table 1), as well as by structural variation in the human genome still being created by L1 activity (Ewing and Kazazian 2010).

**Table 1:** Examples of Transposable Element (TE)-Generated Polymorphic Traits Identified in Inbred Mouse Strains

TE-Generated Trait	Gene Affected	Gene Function	TE Responsible	Mouse Strain	Type of Event	Effect	Tissue Expression	Type of TE-Thrust	Reference
	*Rp2*	GTPase activating protein	B1	DBA	Exonization	Novel isoform	Various	Active	King et al. 1986;
Behavior, pain sensitivity and drug response	*Comt*	Catecholamine neurotransmitter degradation	B2	Various	Exonization	Novel isoform	Brain, various	Active	Li et al. 2010;Kember et al. 2010;Segall et al. 2010;
Fetal survival?	*Psg23*	Pregnancy-specific glycoprotein	LTR	Various	Exonization	Novel isoform	Placenta	Active	Ball et al. 2004;
	*Wiz*	Transcriptional regulation	LTR	C57BL/6, C57BR/cdJ	Exonization	Novel isoform	Various	Active	Baust et al. 2002;
Opioid sensitivity	*Oprm1*	Opioid receptor	ERV	CXBK	Exonization	Novel isoform	Nervous system	Active	Han et al. 2006;
Yellow fur/high body mass	*Agouti*	Pigmentation/energy metabolism	ERV	Yellow obese	Regulatory	Major promoter	Various	Active	Duhl et al. 1994;Morgan et al. 1999;
	*Vipr2*	Vasoactive intestinal peptide receptor	L1	BALB/c	Regulatory	Positive regulation	Various	Active	Steel and Lutz 2007;
	*Alas1*	Nonerythroid heme metabolism	B2	DBA/2	Regulatory	Negative regulation	Various	Active	Chernova et al. 2008;
	*Pcdha*	Neural circuit development	ERV	Various	Regulatory	Positive/negative regulation	CNS	Active	Sugino et al. 2004;
	*Ipp*	Cytoskeleton organization?	LTR	Various	Regulatory	Alternative promoter	Placenta	Active	Chang-Yeh et al. 1993;
Low C4 production	*C4*	Complement factor	B2	Various	Gene disruption	Low expression	Liver	Active	Zheng et al. 1992;
Persistence of alpha-fetoprotein and H19 expression	*Zhx2*	Transcriptional repressor	ERV	BALB/cJ	Gene disruption	Low expression	Liver, various	Active	Perincheri et al. 2005;
White coat spotting	*Ednrb*	Endothelin receptor	Unknown	SSL/LeJ	Gene disruption	Low expression	Various	Active	Yamada et al. 2006

Due to their gaining resistance to recently developed insecticides, and their colonization of new climatic regions, insects provide a good model to study very recent and ongoing realization of adaptive potential due to TE-Thrust in action. The history of the use of insecticides is largely known and the adaptive evolution of resistance is rapid, and has been well studied. There have been multiple recent cases clearly demonstrating a functional link between TE-Thrust and this adaptive change (Chung et al. 2007; Darboux et al. 2007; González et al. 2009, 2010; Schmidt et al. 2010).

A specific example of an adaptive benefit from TE activity is the development of insecticide resistance in the Hikone-R strain of *Drosophila melanogaster*. Three different TEs, apparently involved in four steps, have contributed significantly to the cumulative evolution of resistance to synthetic insecticides, such as DDT, in this strain, with the widespread use of these insecticides commencing in the 1940s (Schmidt et al. 2010). The use of these insecticides allowed a study of the adaptive response to a single environmental component on a timescale that enabled multiple cumulative genetic changes to be observed.

- Step 1. Increased insecticide resistance in the Hikone-R strain was initially derived from an insertion of a 491 bp LTR from an *Accord* retro-TE into the regulatory region of the *Cyp6g1* gene encoding a cytochrome P450 enzyme capable of metabolizing, multiple insecticides, especially DDT (Daborn et al. 2002; Schmidt et al. 2010). This TE insertion, which increases insecticide resistance in this and other strains, is not found in flies collected before 1940, but is now found at high frequency (32–100%) in contemporary *D***.** *melanogaster* populations (Schmidt et al. 2010).
- Step 2. A duplication event yielding two copies of *Cyp6g1* in the Hikone-R strain of *Drosophila*. Possibly, the *Accord* TE insertion and the gene duplication occurred in the one complex event, requiring only one selective sweep to explain the observed rapid increase in insecticide resistance.
- Step 3. The insertion of a *HMS Beagle* TE into the previous insertion derived from the *Accord* LTR.
- Step 4. A partial *P*-element was inserted into the previous insertion derived from the *Accord* LTR, further increasing insecticide resistance. All flies that carry a *P*-element insertion also contain the *HMS Beagle* insertion.

These four steps have occurred within 70 years in the Hikone-R strain of *Drosophila melanogaster*, and the more derived the allele, the greater the resistance (Schmidt et al. 2010). Such allelic successions, whereby different adaptive alleles are substituted sequentially have been demonstrated in several other studies of insecticide resistance (Schmidt et al. 2010).

An example, from another suborder of insects, of the adaptive potential of TEm-alleles is the resistance to a newly encountered natural insecticide, the microbial larvicide *Bacillus sphaericus*. This has as its major active constituent a binary toxin. Resistance in a field-evolved population of the West Nile virus vector, the mosquito *Culex pipiens*, was mediated by a TE insertion into the coding sequence of the midgut toxin receptor gene (*Cpm1*) (Darboux et al. 2007). This induced a new mRNA splicing event, by unmasking cryptic donor and acceptor sites located in this host *Cpm1* gene. The creation of a new intron results in the expression of an altered membrane protein that cannot interact with the toxin, giving an adaptation to environmental contact with this insecticide (Darboux et al. 2007).

The migration of *D. melanogaster* out of sub-Saharan Africa and its adaptation to temperate climates in North America, a few centuries ago and into Australia a century ago, represents another good example of latent adaptive potential due to TEs being realized in a recent real-world context. Various TEs, modifying a diverse set of genes, have apparently played a significant role in adaptation of these flies to temperate climates on both continents (González et al. 2010). At least eight TEm alleles, which were present in low frequencies in the African population, but showed evidence of recent positive selection for adaptation to a temperate climate, were identified. Examples are:

- A solo-LTR inserted into a conserved region of the first intron of the *sra* gene, which critically affects female ovulation and courtship.
- A LINE-like TE inserted in the intergenic region between the *Jon65Aiv* and *Jon65Aiii* genes, both of which have been associated with odor-guided behavior (Anholt and Mackay 2001).
- A LINE-like TE inserted into a circadian regulated gene *CG34353;* (González et al. 2010).

## A Partial Unification of Empirically Derived TE-Thrust Data with more Theoretically Derived Syntheses

The latent adaptive potential of the alleles of the genes above, the *sra* gene, the *Jon65Aiv* and *Jon65Aiii* genes, and the *CG34353* gene were realized in colonization of new areas. These TEm-alleles are adaptive for the colonization of temperate climates by *D. melanogaster*, and are present in low frequencies in the original sub-Saharan African population (González et al. 2010) where they were not adaptive, but were only potentially adaptive in a changed environment or ecosystem. Their presence in sub-Saharan African populations demonstrates latent adaptive potential, or standing variation, due to TE-Thrust. The realization of this adaptive potential by rapid positive selection of these TEm-alleles, coinciding with the expansion of the flies into temperate areas, is a change in allele frequencies, as is proposed in modern evolutionary syntheses. Thus, in this respect, the TE-Thrust hypothesis and the Modern Synthesis are in agreement.

## The Failure of Mutation Breeding

In a review, Lönnig (2005), described how, despite early enthusiasm and sustained effort, mutation breeding (in either plants or animals) has never been successful. The mutations caused by mutagens usually produced weaker or nonfunctional alleles of wild type genes. In TE-Thrust, however, the TEs usually consist of functional coding or exaptable sequences, and often also of potent regulatory sequences, so that by insertion and in many other ways, for example, exon shuffling in the active mode and ectopic recombination in the passive mode, they can make many beneficial changes, although they may sometimes do damage (Oliver and Greene 2009a,b, 2011). TEs can alter the regulation or the structure of alleles, or duplicate them (Darboux et al. 2007; González et al. 2009, 2010; Schmidt et al. 2010) creating TEm-alleles. Therefore, although attempted breeding, adaptation or evolution, using mutagens to generate alternative alleles almost always does not work (Lönnig 2005), adaptation or evolution using TE-Thrust generating TEm-alleles relatively often does work. This is not to say that other types of mutation, such as point changes, are not important in evolution. In fact, in addition to their general importance in evolution, such mutations often complement TE-Thrust, for example, by modifying TE-duplicated sequences.

## Reduced “Fitness” versus Enhanced “Evolutionary Potential”

The question of whether or not the possible lowering of fitness in a lineage by TEs can result in enhanced evolutionary potential may be simplified into two competing hypotheses:

*The Null Hypothesis*: TE-Thrust is not causal to adaptation, speciation, punctuation events, or evolution.

*The Alternative Hypothesis*: TE-Thrust is causal to adaptation, speciation, punctuation events, and evolution.

## Testing the Hypotheses

### Recent/ancient speciation and the alternative (TE-Thrust) hypothesis

In the absence of events, such as intermittent *de novo* modifications to successive families of TEs, *de novo* SINE synthesis, HTT, or *de novo* synthesis of chimaeric TE elements, TE bursts in lineages eventually tend to fade to inactivity, with TEs becoming nonviable and degraded by the accumulation of deleterious mutations. An example is the apparent loss of L1 element activity in a number of species. These include the spider monkey, thirteen-lined ground squirrel, megabats, and sigmodontinae rodents (Casavant et al. 2000; Boissinot et al. 2004; Cantrell et al. 2008; Platt and Ray 2012), although at least in the case of the sigmodontinae, which have undergone rapid fecund speciation with numerous karyotypic changes, the loss of viable LINEs appears to have been more than compensated for by massive endogenisations of ERVs (Cantrell et al. 2005; Erickson et al. 2011). As TE-Thrust predicts that lineages lose their adaptability as overall TE activity and integrity fades, the loss of TE viability over time provides an intragenomic explanation to help account for the high rate of background extinction that has been a prevalent feature of life on earth (Raup 1994). In contrast, lineages harboring young TE families are associated with recent speciation. This is well exemplified in the mammals where species with the highest numbers of young TE families, such as the mouse, rat, bat, rhesus macaque, and human, represent the largest extant mammalian orders of rodents, bats, and primates (Jurka et al. 2011). Very species-poor extant mammalian lineages, such as the alpaca, elephant, tenrec, armadillo, and platypus, do not harbor any young families of TEs (Jurka et al. 2011). Nevertheless, TE-Thrust predicts more ancient speciation events being attributed to older families of TEs, when they were young, and this is supported by phylogenetic analyses (Jurka et al. 2011). These data are consistent with the Alternative (TE-Thrust) Hypothesis.

### The vesper bats and the alternative (TE-Thrust) hypothesis

The radiation of the vesper bats (family Verspertilionidae) appears to support the *Alternative Hypothesis* and the active mode of TE-Thrust. The vesper bats, which have an almost worldwide distribution (Nowak 1994), are a fecund lineage (407 species of the approximately 930 species of microbats or 8–9% of all extant mammal species), and include *Myotis*, the most speciose mammalian genus with about 103 members. Significantly, vesper bats are somewhat unique in having many viable and active DNA-TEs that have been nonviable in most other mammals for 37 Myr (Pace and Feschotte 2007).

- The early radiation of the vesper bats is proposed to have been due to HTT of *Helitron* DNA-TEs, called *Helibat,* into the vesper bat lineage about 30–36 Mya (Pritham and Feschotte 2007).
- Amplification of DNA-TEs is thought to follow HTT in a naive lineage, which can result in innovations in the genome (Pace et al. 2008).
- *Helibat* has amplified explosively up to at least 3.4% of the *Myotis lucifugus* genome (Ray et al. 2008).
- HTT of Helitrons, especially, can lead to diversification, and to dramatic shifts in the trajectory of genome evolution (Thomas et al. 2010).
- HTT of of DNA-TEs can also lead to horizontal gene transfer (Thomas et al. 2010).
- Although Helitrons have not been detected in other mammals besides the vesper bats, they are abundant in plants, invertebrates, and zebrafish, and have been implicated in large-scale gene duplication and exon shuffling.
- There were other multiple waves of HTT of DNA-TEs in the bat lineage coinciding with a period of their rapid diversification 16–25 Mya (Teeling et al. 2005; Pritham and Feschotte 2007; Ray et al. 2008).
- A further burst of New World *Myotis* diversification 12–13 Mya was noted (Stadelmann et al. 2007), corresponding well with the period that the most active transposition of a variety of DNA-TEs is estimated to have occurred (Ray et al. 2008).
- Such repeated waves of TE activity suggest a mechanism for generating the genetic diversity needed to result in the evolution of such great species richness as is observed in the vesper bats (Ray et al. 2008).
- Active retro-TEs, namely L1 LINEs (Cantrell et al. 2008) and VES SINEs (Borodulina and Kramerov 1999), have also been found in vesper bats.

This mix of viable DNA-TEs and retro-TEs, unknown in other mammals, could have resulted in large architectural and organizational changes in their genomes and aided in the *Myotis* diversification, enabling adaptation to very diverse ecological niches within this lineage (Pritham and Feschotte 2007; Thomas et al. 2011). This suggests that much active TE-Thrust has operated during the very large radiation of the vesper bats during the last 36 Myr. A lack of data presently obscures any conclusions regarding any possible involvement of passive TE-Thrust. The predicted evolutionary outcome of such intermittently active populations of TEs is either gradualism or stasis with punctuation events, (Type I or II punctuated equilibrium). Current data suggest that this is correct for the Verspertilionidae.

### The Muridae Rodents and the Alternative (TE-Thrust) Hypothesis

The extensive radiation of the Old World Muridae (the Murinae) appears to support the *Alternative Hypothesis*, and both the active and the passive modes of TE-Thrust. The rodents are the most fecund mammalian order comprising about 40% of mammals with an almost worldwide distribution. The Muridae family, which include the true mice and rats, have been particularly successful and account for about two-thirds of all rodent species. Representatives of the subfamily Murinae (*Mus* and *Rattus*) possess large populations of relatively homogenous retro-TEs, many of which are viable and active (Table 2).

- The Old World mouse (*Mus*) and rat (*Rattus*), with some 50–60 species each in their respective genera, have genomes comprised of about 40% largely homogenous genomic TEs. These include numerous viable and mostly highly active L1 LINEs and few nonviable ancient L2 LINEs, giving a LINE total of 22%. SINEs comprise a further 7% and most (92%) are lineage specific, viable, and effective, although slightly diverse, with only few being the nonviable ancient MIR SINEs. Less than 1% of their genomes are composed of nonviable DNA-TEs (Waterston et al. 2002; Gibbs et al. 2004). The mouse has about 10% ERV/sLTRs, many of which are very active and are closely related to mouse exogenous retroviruses (Maksakova et al. 2006).
- The fitness cost of their greatly enhanced evolutionary potential is higher than in humans, as previously noted (Maksakova et al. 2006).

**Table 2:** Presence and Viability of Transposable Elements (TEs) in Distinct Mammalian Species

	Human	Mouse	Naked Mole Rat	Platypus
Genome Size (Gbp)	3.1	2.6	2.7	2.3
TE Content (% genome)	45.5	40.9	25	44.6
LINE	**Some viable (LINE1)**	**Some viable (LINE1)**	Nonviable	Some possibly viable (mainly ancient LINE2)
SINE (Lineage-specific)	**Some viable (Alu, SVA)**	**Some viable (*****e.g.,*** **B1, B2)**	Nonviable	Rare/absent
SINE (Widespread)	Nonviable	Nonviable	Nonviable	Some possibly viable (mainly ancient MIR/Mon-1)
LTR/ERV	Some possibly viable	**Some viable**	Nonviable	Rare (LTR), absent (ERV)
DNA-TE	Nonviable	Nonviable	Nonviable	Rare

Although the generally small size of many rodents probably aided in their diversification, there has seemingly been much active TE-Thrust, as indicated by the growing number of documented examples of rodent-specific traits generated by TEs (Table 3). They are also quite well suited to passive TE-Thrust, as they have large homogenous populations of TEs to facilitate TE-mediated duplications, inversions, deletions or karyotypic changes. The predicted evolutionary outcome of large homogenous and intermittently active populations of TEs is gradualism with punctuation events (Type II punctuated equilibrium), as in the hypothesized mode 2 of TE-Thrust.

**Table 3:** Specific Examples of Transposable Elements (TEs) Implicated in Rodent-Specific Traits

TE-Generated Trait	Gene Affected	Gene Function	TE Responsible	Distribution	Type of Event	Effect	Tissue Expression	Type of TE-Thrust	Reference
	*Mtfull*	Unknown	LTR	>Mouse	Domestication	Novel gene	Ovary	Active	Holt et al. 2006;
Placental morphogenesis	*Syncytin-A*	Trophoblast cell fusion	ERV	Muridae	Domestication	Novel gene	Placenta	Active	Dupressoir et al. 2005;
Placental morphogenesis	*Syncytin-B*	Trophoblast cell fusion/immunosuppression	ERV	Muridae	Domestication	Novel gene	Placenta	Active	Dupressoir et al. 2005;Mangeney et al. 2007;
Virus resistance	*Fv1*	Blocker of retrovirus replication	ERV	Mus	Domestication	Novel gene		Active	Bénit et al. 1997;
	*Soro-1*	Unknown	ERV	Rat	Domestication	Novel gene	Heart, liver	Active	Martin et al. 1995;
	*Tyms*	Thymidylate synthetase	L1	>Mouse	Exonisation	Major isoform	Various	Active	Harendza and Johnson 1990;
	*Pphln1*	Epithelial differentiation/nervous system development	SINE/LTR	>Mouse	Exonization	Novel isoforms	Fetal, various	Active	Huh et al. 2006;
Soluble LIFR	*Lifr*	Cytokine receptor	B2	Mouse	Exonization	Novel isoforms	Various	Active	Michel et al. 1997;
	*H2-d*	Antigen presentation to the immune system	B2	Mouse	Exonization	Novel isoform	Various	Active	Kress et al. 1984;
	*H2-l*	Antigen presentation to the immune system	B2	Mouse	Exonization	Novel isoform	Various	Active	Kress et al. 1984;
	*Phkg1*	Glycogen catabolism	B2	>Mouse	Exonization	Novel isoform	Muscle, various	Active	Maichele et al. 1993;
	*Tdpoz-T1*	Regulation of protein processing and ubiquitination?	L1/ERV/SINE1/hAT	>Rat	Exonization	Novel isoforms	Testis, embryo	Active	Huang et al. 2009;
	*Tdpoz-T2*	Regulation of protein processing and ubiquitination?	L1/ERV	>Rat	Exonization	Novel isoforms	Testis, embryo	Active	Huang et al. 2009;
	*Pmse2*	Proteasome activator	L1	>Mouse	Regulatory	Major promoter	Various	Active	Zaiss and Kloetzel 1999;
	*Ocm*	Calcium binding protein and growth factor	LTR	>Rat	Regulatory	Major promoter	Macrophage	Active	Banville and Boie 1989;
	*Naip*	Anti-apoptosis	LTR	>Muridae	Regulatory	Major/alternativepromoter	Various	Active	Romanish et al. 2007;
	*Mok-2*	Transcription factor	B2	>Mouse	Regulatory	Negative regulation	Brain, testis	Active	Arranz et al. 1994;
	*Igk*	Immunoglobulin light chain	B1	>Mouse	Regulatory	Negative regulation	B cell	Active	Saksela and Baltimore 1993;
	*SINE/B1 small RNAs*	Embryonic postranscriptional gene silencing?	B1	>Mouse	Regulatory	Negative regulation	Embryo	Active	Ohnishi et al. 2012;
	*Ins1*	Insulin	LINE	>Rat	Regulatory	Negative regulation	Pancreas	Active	Laimins et al. 1986;
	*EpoR*	Erythropoietin receptor	Unknown	>Mouse	Regulatory	Negative regulation	Erythroid	Active	Youssoufian and Lodish 1993;
	*Gh*	Growth hormone	B2	>Mouse	Regulatory	Insulator element	Pituitary gland	Active	Lunyak et al. 2007;
	*Slp*	Complement activity?	ERV	>Mouse	Regulatory	Androgen responsiveness	Liver, kidney	Active	Stavenhagen and Robins 1988;
	*Lama3*	Cell attachment, migration and organization	B2	>Mouse	Regulatory	Alternative promoter	Various	Active	Ferrigno et al. 2001;
	*Nkg2d*	NK and T cell activating receptor	B1	>Muridae	Regulatory	Alternative promoter	NK/T cells	Active	Lai et al. 2009;
Cell growth control?	*s-myc/ms-myc*	Unknown	Unknown	>Muridae	Retrotransposition	Novel gene	Embryo	Active	Sugiyama et al. 1999;
	*N-myc2*	Unknown	Unknown	>Sciuridae	Retrotransposition	Novel gene	Brain	Active	Fourel et al. 1992;
	*Zfa*	Unknown	Unknown	>Mouse	Retrotransposition	Novel gene	Testis	Active	Ashworth et al. 1990;
Efficient energy utilization?	*Ins1*	Insulin	Unknown	Old World Rats and Mice	Retrotransposition	Novel gene	Pancreas	Active	Soares et al. 1985;
	*Pabp2*	mRNA regulation	Unknown	>Mouse	Retrotransposition	Novel gene	Testis	Active	Kleene et al. 1998;
	*Amd2*	Polyamine biosynthesis	Unknown	>Mouse	Retrotransposition	Novel gene	Liver, various	Active	Persson et al. 1995;
	*G6pd2*	Pentose phosphate pathway enzyme	Unknown	Mouse	Retrotransposition	Novel gene	Testis	Active	Hendriksen et al. 1997;
	*Pem2*	Transcription factor	Unknown	>Rat	Retrotransposition	Novel gene	Epididymis	Active	Nhim et al. 1997;
	*Phgpx*	Antioxidant defense, spermatogenesis	Unknown	>Mouse	Retrotransposition	Novel gene	Various	Active	Boschan et al. 2002;
	*Arxes1/2*	Adipogenesis	Unknown	>Rodent	Retrotransposition	Novel gene	Adipose tissue	Active	Prokesch et al. 2011;
	*Mrg(s)*	Nociceptive neuron function	L1	>Mouse	Duplication	Novel genes	Sensory neurons	Passive	Zylka et al. 2003

> = Maximum known distribution.

### The naked mole rat and the alternative (TE-Thrust) hypothesis

In sharp contrast to *Mus* and *Rattus*, which are both very rich in species and have abundant viable and active TEs (Waterston et al. 2002; Gibbs et al. 2004), the rodent genus *Heterocephalus,* also in the family Muridae, has only one species (Wilson and Reader 2005). In support of the *Alternative Hypothesis*, sequencing of *H. glaber* (Kim et al. 2011)*,* the very atypical, physiologically unique, eusocial, and long-lived naked mole rat, has shown that it possesses a nonviable and relatively small mobilome consortium (Table 2).

- The TEs of the naked mole rat, although they are homogenous and constitute 25% of the genome, are highly divergent, indicating they have been both nonviable and inactive for a very long time (Kim et al. 2011).
- As most mammals have 35–50% TEs, this suggests that a substantial portion of its TEs may have been lost altogether.

The data indicate that *H. glaber* has had little or no TE-Thrust, except in the remote past, and if all else is equal, it is in stasis or gradualism. (Note: As viable and active TEs are known to occasionally cause harmful mutations, these data additionally suggest that there possibly could be less genetic disease and cancer in the individuals of species, such as *H. glaber*).

### The platypus and the alternative (TE-Thrust) hypothesis

Although microbats and rodents may owe some of their diversity of species to their small size, the monotremes are also rather small animals, so size would not appear to be a major factor in their lack of radiation, with just some three species (Pough et al. 2009), including only one extant species of platypus. Although a large fraction of the platypus genome consists of TEs, the fact that these are largely ancient and inactive (Table 2) appears to support the *Alternative Hypothesis*.

- About 50% of the platypus genome is derived from TEs, but these consist of about 1.9 million severely truncated copies of the ancient L2 LINEs, only some of which are putatively viable, and 2.75 million copies of the ancient SINE MIR/Mon-1, which became extinct (nonviable) in marsupials and eutherians 60–100 Mya (Warren et al. 2008).
- The platypus possesses few DNA-TEs and LTR retro-TEs, but there are copies of an ancient *gypsy*-class LTR retro-TE (Warren et al. 2008).
- There are apparently no ERV/sLTRs (Warren et al. 2008).
- There have seemingly never been any notable infiltrations by ERVs, or HTT of DNA-TEs. This appears significant given the aforementioned importance of retroviruses to the placenta, as well as given the critical role that DNA-TEs appear to have had in generating gene regulatory networks that underlie the ability of the uterine endometrium to accommodate pregnancy via embryonic implantation (Lynch et al. 2011).
- The platypus seems to never have had the L1 LINEs, or Bov-B LINEs, of most mammals, and has apparently never had lineage-specific SINEs, such as the Alu of simians, or the B1 of rodents.
- Platypus evolution has been extremely conservative, especially in tooth form and body size, for 120 Myr (Flannery 1994).

Although the platypus has an abundance of a restricted range of some ancient, and seemingly mostly nonviable TEs, there appears to have been very little active TE-Thrust in the platypus genome in a long time. These data clearly suggest support for the alternative hypothesis above. According to the TE-Thrust hypothesis, the platypus should support some passive TE-Thrust due to its large, but mostly nonviable, homogeneous TE consortium. The predicted evolutionary outcome of a large homogenous population of mostly nonviable TEs, is gradualism, as in the hypothesized mode 4 of TE-Thrust. This, from current data, appears to be correct for the platypus.

### The green anole lizard, the tuatara, and the alternative (TE-Thrust) hypothesis

The *Anolis* clade of lizards comprises some 400 species that have radiated extensively in the Neotropics. In support of the *Alternative Hypothesis*, sequencing of one species (*Anolis carolinensis*) has shown that its genome possesses multiple young and highly active retro-TE and DNA-TE families (Alföldi et al. 2011).

- The genome of the green anole lizard, *A. carolinensis,* contains about 30% active TEs, with about 8% being comprised of a variety of LINEs (L1, L2, CR1, RTE, and R4) that seem to be recent insertions based on their sequence similarity (Novick et al. 2009; Alföldi et al. 2011). Another 5.3% of the genome are SINEs.
- DNA-TEs are young and diverse, with at least 68 families belonging to five superfamilies, hAT, Chapaev, Maverick, Tc/Mariner, and Helitron (Novick et al. 2011).

The green anole lizard has an extremely wide diversity of active TE families, with a low rate of accumulation, similar to the TE profile of teleostean fishes (Novick et al. 2009; Alföldi et al. 2011). Thus, active TE-Thrust appears to be strongly implicated as a significant factor in the major radiation of this lineage of lizards. A large heterogeneous consortium of intermittently active TEs is hypothesized to result in stasis with intermittent punctuation events (type I punctuated equilibrium), as in Mode 1 of the TE-Thrust Hypothesis.

The green anole lizard contrasts with the two lizard-like “living fossil” species of the tuatara, which have a paucity of TEs estimated to be less than 3% (Wang et al. 2006), and that, so far, as is known, appear to be nonviable (Kapitonov and Jurka 2006). The stark difference in TE consortia between these species points to an almost complete lack of TE-Thrust in the tuatara consistent with evolutionary stasis. This appears to support the Alternative (TE-Thrust) Hypothesis.

### Reproductive isolation and speciation and the alternative (TE-Thrust) hypothesis

Reproductive isolation, which is generally considered to be a prerequisite for speciation, has been attributed to the division of a population into demes (Wright 1931; Eldredge 1995; Jurka et al. 2011). Speciation has also been associated with the availability of occupiable niches, and we agree that this can be a contributing factor. However, as highlighted below, karyotypic changes due to the presence and activity of TEs may also be an important factor in reproductive isolation and speciation.

- The order Rodentia originated >57 Mya. The family Muridae contains an extraordinary 26% of extant mammalian species and evolved only about 20 Mya.
- Karyotypic changes between the Old World mouse and rat, representing the very speciose *Mus* and *Rattus* genera (Muridae: subfamily Murinae) have proceeded 10 times faster than that between humans and cats (Stanyon et al. 1999). The Old World mouse and rat have 23 and 21 young families of TEs (<1% divergence from the consensus sequence) with total counts of inserted TEs in these young families of 1,930 and 5,755, respectively, (Jurka et al. 2011) indicating much recent TE activity.
- The very large recent radiation of some New World rodents (Muridae: subfamily Sigmodontinae) has been coincident with extreme karyotypic variation between species (Grahn et al. 2005) and with extraordinarily numerous ERV (MysTR) endogenizations, (Cantrell et al. 2005; Erickson et al. 2011).
- The sole extant species of the platypus represents a lineage that has been extremely conservative in its evolution during its 120 Myr history, even between Australian and South American (fossil) species (Flannery 1994). The extant platypus has no young TE families with <1% divergence from the consensus sequence (Jurka et al. 2011), so has had apparently had no recent TE activity, suggesting a lack of a causal agent for karyotypic changes and speciation.

### Summary of the evidence for the alternative (TE-Thrust) hypothesis

It can, of course, be argued that this evidence in mammals (microbats, rodents, and the platypus), reptiles (the green anole lizard and the tuatara), and the evolution of the mammalian placenta, is all only circumstantial evidence, and therefore does not demonstrate a causal link between TE-Thrust and enhanced evolutionary potential. This argument is weakened by the abundance of young families of TEs in the largest extant mammalian orders of rodents, bats, and primates, and their absence in the elephant, alpaca, tenrec, armadilo, and platypus. The argument of “only circumstantial evidence” is further weakened by the wide range of known conserved and/or beneficial genomic modifications that are due to TEs in various lineages (Brosius 1999; Miller et al. 1999; Kidwell and Lisch 2001; Nekrutenko and Li 2001; van de Lagemaat et al. 2003; Jordan et al. 2003; Kazazian 2004; Shapiro and Sternberg 2005; Volff 2006; Böhne et al. 2008; Oliver and Greene 2009a, 2011). Therefore, it seems that a causal link between recent TE activity, sometimes resulting in reproductive isolation, and recent speciation events is indeed likely.

Some hard evidence can be provided with regard to adaptive potential and adaptive evolution in insecticide resistance by insects in the last 70 years, and adaptation to temperate climates in the last few centuries. However, a punctuation event is estimated to take between 15,000 and 40,000 years (Gould 2002). It appears then that, as yet, bursts of TE activity and punctuation events cannot be dated accurately enough to establish any definite relationship. However, some apparent correlations have been reported, suggesting that increased TE activity may indeed be basal to, or coincident with, punctuation events and evolutionary transitions, speciation, or large radiations. Some examples of these, in addition to those detailed above, are:

- Ohshima et al. (2003) found bursts of Alu SINE and retrocopies coincident with the radiation of the higher primates 40–50 Mya.
- DNA-TE activity coincided with speciation events in salmonoid fishes (de Boer et al. 2007).
- Bursts of transposition of *BS* element transposition have also shaped the genomes of at least two species of *Drosophila, D. mojavensis* and *D. recta* (Granzotto et al. 2011).
- There are numerous examples of bursts of TE activity that often follow polyploidization events (Comai 2000), or hybidization (Michalak 2010), in angiosperms, leading to speciation.

Some suggest that a role for TEs in speciation is speculative (Hua-Van et al. 2011), whereas others have given data, which they readily acknowledge specifically suggests TE involvement in taxon radiations (de Boer et al. 2007; Pritham and Feschotte 2007; Ray et al. 2008; Thomas et al. 2011). In our interpretation of the available data, we suggest that, if all else is equal, minimal or passive TE-Thrust is likely to result in stasis or gradualism, whereas active TE-Thrust is likely to be causal to innovative evolution (e.g., the placenta), punctuation events and radiations, as in our hypothesized four modes of TE-Thrust (Oliver and Greene 2011). However, we readily acknowledge that some punctuation events may be caused by other facilitators of evolution.

## Conclusions

The field of evolutionary biology has seemingly paid more attention to the outcomes of genetic mutation in terms of the generation of variants and their selection within populations than the mechanisms by which mutations emerge in the first place. Although small-scale DNA base changes and deletions are important in evolution, TEs (and viruses) are uniquely placed, via TE-Thrust, to expeditiously cause complex and/or large-scale changes and thereby help explain macroevolutionary change and the emergence of highly innovative adaptations. Much still remains to be investigated, such as the relevance of TE-Thrust to other classes and phyla. Only a small number of lineages in the metazoans: the mammals and to a lesser extent, a very few lineages of the insects, plants, and reptiles, have been considered with regard to the TE-Thrust hypothesis to date. As increasing numbers of genomes are being sequenced, it would be interesting to investigate the link between TEs, exogenous viruses, and enhanced adaptive potential, enhanced evolutionary potential, evolutionary transitions, and the occurrence of punctuation events, in the lineages of other taxa. It seems likely that in the great diversity of extant lineages, TE-Thrust and other facilitators of evolution will have had a greater or lesser impact on adaptation and evolution. There seems to be little doubt, however, that TEs and viruses have played a major and prominent role in the evolution of almost all life on earth, and that TEs and viruses need to be recognized and included, as the TE-Thrust hypothesis, in a much needed extension and modification in evolutionary theory.
